# Prevalence of nontuberculous mycobacteria in a tertiary hospital in Beijing, China, January 2013 to December 2018

**DOI:** 10.1186/s12866-020-01840-5

**Published:** 2020-06-12

**Authors:** Jing-jing Huang, Ying-xing Li, Ying Zhao, Wen-hang Yang, Meng Xiao, Timothy Kudinha, Ying-chun Xu

**Affiliations:** 1 Department of Clinical Laboratory, Peking Union Medical College Hospital, Peking Union Medical College, Chinese Academy of Medical Sciences, Beijing, 100730 China; 2 Beijing Key Laboratory for Mechanisms Research and Precision Diagnosis of Invasive Fungal Diseases, Beijing, 100730 China; 3 Graduate School, Peking Union Medical College, Chinese academy of Medical Science, Beijing, 100730 China; 4 Department of Medical Research Center, Peking Union Medical College Hospital, Chinese Academy of Medical Science & Peking Union Medical College, Beijing, 100730 China; 5 Charles Sturt University, Leeds Parade, Orange, Sydney, NSW Australia; 6 Centre for Infectious Diseases and Microbiology Laboratory Services, ICPMR-Pathology West, Westmead Hospital, Westmead, NSW Australia

**Keywords:** Mycobacterium, Nontuberculous mycobacteria, Identification, Gene chip

## Abstract

**Background:**

To investigate the species distribution of non-tuberculous mycobacteria (NTM) among tuberculosis (TB) specimens collected from January 2013 to December 2018 at Peking Union Medical Hospital (Beijing), China. NTM species identification was carried out by DNA microarray chip.

**Results:**

Mycobacterial species were detected in 1514 specimens from 1508 patients, among which NTM accounted for 37.3% (565/1514), increasing from a proportion of 15.6% in 2013 to 46.1% in 2018 (*P* < 0.001). Among the 565 NTM positive specimens, the majority (55.2%) were from female patients. Furthermore, patients aged 45–65 years accounted for 49.6% of the total patients tested. Among 223 NTM positive specimens characterized further, the majority (86.2%) were from respiratory tract, whilst 3.6 and 3.1% were from lymph nodes and pus, respectively. *Mycobacterium intracellulare* (31.8%) and *Mycobacterium chelonae* / *Mycobacterium abscessus* (21.5%) were the most frequently detected species, followed by *M. avium* (13.5%), *M. gordonae* (11.7%), *M. kansasii* (7.6%), and others.

**Conclusion:**

The proportion of NTM among mycobacterial species detected in a tertiary hospital in Beijing, China, increased rapidly from year 2013 to 2018. Middle-aged patients are more likely to be infected with NTM, especially females. *Mycobacterium intracellulare* and *Mycobacterium chelonae*/ *Mycobacterium abscessus* were the most frequently detected NTM pathogens. Accurate and timely identification of NTM is important for diagnosis and treatment.

## Background

The substantial increase in the number of patients with immunodeficiency disease in recent years has contributed to the rise in infectious diseases caused by a variety of rare organisms, including non-tuberculous mycobacteria (NTM) [1, 2]. In China, patients diagnosed with or suspected of having tuberculosis (TB), are referred to a thoracic specialist hospital for further treatment. The clinical manifestations of TB and some NTM species infections are similar [3], with a considerable number of patients with putative NTM infections lost to follow up in tertiary hospitals. In addition, the treatment strategies used for management of infections caused by various NTM species are significantly different from those of TB [4]. Thus accurate identification of *Mycobacteria* strains to species level is crucial for managing infections [5].

The distribution of NTM species in different clinical settings has been investigated by chest and/or TB hospitals in some provinces and regions of China [6, 7]. However, data on these organisms from tertiary hospitals in China is limited. In 2013, the Non-tuberculous Mycobacteria Network European Trials Group (NTM-NET) reported on the geographic diversity of NTM species isolated from pulmonary samples, using data on isolates collected from 30 countries across six continents [8]. The group found out that *Mycobacterium avium* complex (MAC) was the most frequently isolated species in most countries (e.g. Australia, Japan, and 13 countries in Europe), followed by *M. gordonae* and *M. xenopi*. However, this large study was not able to collect data from China.

Since 2013 to date, the Microbiology lab at Peking Union Medical College hospital has been using PCR-fluorescence probe method (Tuberculosis and Non-Tuberculous Mycobacteria Real-time PCR Detection Kit, CapitalBio Technology Inc., Beijing, China) to directly detect tuberculous/non-tuberculous *Mycobacterium* in clinical samples. DNA microarray chip method (Mycobacterial Species Identification Array Kit, CapitalBio Technology Inc., Beijing, China) was used to identify the different NTM species. These detections are crucial in the early diagnosis of patients with NTM infections. This study retrospectively analyzed the identification data of NTM samples from 2013 to 2018, to provide a general outline of the prevalence of non-tuberculous mycobacteria in a tertiary hospital in China.

## Results

### Changes of MTB/NTM detection rates

**Fig. 1:**
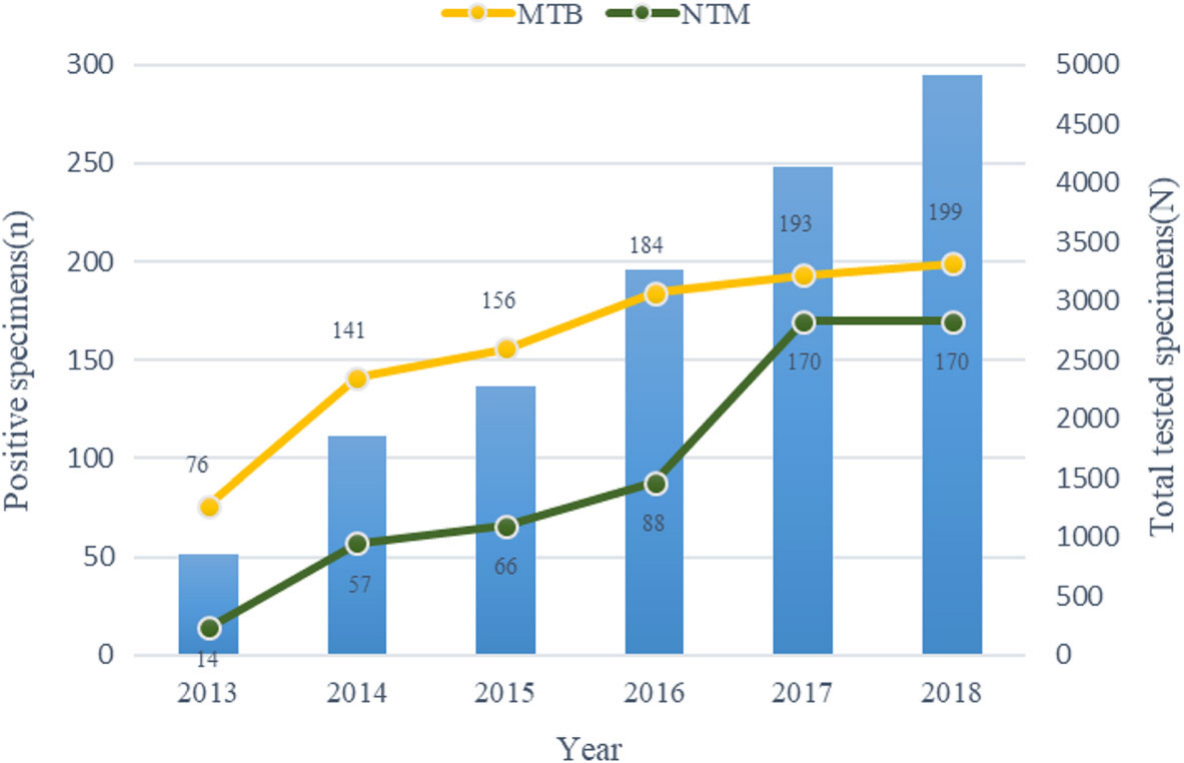
Distribution of Positive specimens in *Mycobacterium* nucleic acid detection during 2013–2018. MTB: *Mycobacterium tuberculosis*; NTM:non-tuberculous mycobacteria.

From 2013 to 2018, 17,287 non-repeat clinical specimens from Peking Union Medical College Hospital were sent to the Microbiology lab for detection of MTB/NTM by PCR-fluorescence probe method (Tuberculosis and Non-Tuberculous Mycobacteria Real-time PCR Detection Kit, CapitalBio Technology Inc., Beijing, China). Of these, 1514 (8.76%) specimens were positive for MTB/NTM (Fig. 1). During the 6 year study period, there was a significant increase in the number of samples tested for TB and/or NTM each year, with a gradual decrease in MTB detection, and a corresponding significant rise in NTM detection, year by year (χ^2^ = 21.77, < 0.001), as shown in Fig. 1. Thus altogether there were 1514 positive specimens obtained from 1508 patients, and 6 patients had mixed infection of MTB and NTM. MTB was detected in 949 (62.7%) of the 1514 positive samples, and NTM in 565 (37.3%). From 2013 to 2018, there was a significant rise in the proportion of NTM, and a corresponding decrease in the proportion of MTB (χ^2^ = 58.84,*P* < 0.001).

### Demographic data of NTM positive patients

The 565 NTM positive patients comprised of 44.8% (253/565) males and 55.2% (312/565) females. The ages of the patients ranged from 10 to 95 years, with an average age of 51.55 ± 16.84 years, and the quartiles were 40, 53 and 63 years, respectively. Only 17 patients (17/565, 3.0%) were under 18 years old. Patients aged 18–44 years accounted for 28.3% (160/565) of the patients, those aged 45–65 years for 49.6% (280/565), and those over 65 years of age accounted for 19.1% (108/565) of the total patients.

### Types of NTM positive specimens

The 565 NTM positive samples were collected from different clinical departments. Most of them (348/565, 61.6%) were from the respiratory department, followed by infection department (55/565, 9.7%), and other departments were relatively less represented (no one was more than 4.4%). According to sample type category, the majority was sputum (243/565, 43.0%), followed by broncho-alveolar lavage fluid (101/565, 17.9%) and tracheobronchial aspiration (74/565, 13.1%), cerebrospinal fluid (69/565, 12.2%), lymph nodes (19/565, 3.4%), pus and urine (both 14/565, 2.5%). Other types of specimens were less than 1.0%. The total number of specimens from the respiratory tract was 418 (74.0%).

### NTM species distribution

Based on the requesting physician’s requirements for further testing, 223 NTM positive samples were characterized for NTM species, with repetitive isolates from the same body part of the same patient excluded. These 223 samples were collected from 220 patients. Among them, two cases were multiple location infection, and one case was mixed infection of *M. intracellulare* and other *Mycobacterium* spp. isolate which was beyond the detection range of our kit and thus could not be identified to species level accurately. The NTM species identified by DNA microarray chip in the 223 samples included *M. intracellulare* (71), *M. chelonae/M. abscessus* (48), *M. avium* (30), *M. gordonae* (26), *M. kansasii* (17), *M. fortuitum* (15), *M. xenopi* (2), *M. gilvum* (2), *M. smegmatis* (1), *M. marinum/M. ulcerans* (1), *M. terrae* (1), *M. phlei* (1) and other *Mycobacterium* spp. (8). In addition, 7 specimens initially identified as other *Mycobacterium* spp. as they could not be identified to species level by Mycobacterial Species Identification Array Kit (CapitalBio Technology Inc., Beijing, China), were further identified as *M. simiae* (2), *M. iranicum*, *M. chimaera*, *M. marswillense*, *M. holsaticum* and *M. colombiense* by 16S rDNA sequencing. Distribution of NTM species identified by DNA microarray chip method during the period 2013–2018, is shown in Table 1.

**Table 1:** Non-tuberculous species identified from 2013 to 2018 in a tertiary hospital in Beijing, China

Gene chip identified *Mycobacterial* Species	2013	2014	2015	2016	2017	2018	Total (%)
*M. intracellulare*	2	2	8	8	20	31	71 (31.8)
*M. chelonae/M. abscessus*	2	3	11	13	10	9	48 (21.5)
*M. avium*	0	1	5	2	6	16	30 (13.5)
*M. gordonae*	0	1	2	3	8	12	26 (11.7)
*M. kansasii*	0	0	2	6	4	5	17 (7.6)
*M. fortuitum*	0	0	1	2	4	8	15 (6.7)
*M. gilvum*	0	0	0	0	1	1	2 (0.9)
*M. xenopi*	0	0	0	0	1	1	2 (0.9)
*M. marinum/M. ulcerans*	0	1	0	0	0	0	1 (0.4)
*M. smegmati*	0	0	1	0	0	0	1 (0.4)
*M. terrae*	0	0	0	0	0	1	1 (0.4)
*M. phlei*	0	0	0	0	0	1	1 (0.4)
Other *Mycobacterium*	0	0	0	2	1	5	8^a^ (3.6)
Total	4	8	30	36	55	90	223 (100)

^a^Seven of the isolates were further identified as *M. simiae* [2], *M. iranicum*, *M. chimaera*, *M. marswillense*, *M. holsaticum* and *M. colombiense* by 16S rDNA sequencing respectively

### NTM species distribution from different specimen types

As shown in Table 2, there are several differences in sample sources for different NTM species. Besides the respiratory tract which was a major site for all NTM species, 45.5% (5/11) of *M. chelonae/M. abscessus* and 50.0% (2/4) of *M. kansasii* were detected from lymph node specimens. Also, there were 33.3% (2/6) and 27.3% (3/11) of *M. intracellulare* and *M. chelonae/M. abscessus* detected from pus, respectively.

**Table 2:** Specimen types among which NTM were identified in this study

Gene chip identified *Mycobacterial* Species	Sputum	Bronchoalveolar lavage fluid	Tracheobronchial aspiration	Lymph node	Pus	Hydrothorax	Urine	Lung tissue	Ascitic fluid	Marrow	Joint fluid	Subcutaneous nodule	Vertebrae puncture tissue	Others	Total
*M. intracellulare*	46	12	7	0	2	1	0	0	0	1	1	1	0	0	71
*M. chelonae/M. abscessus*	35	1	1	5	3	1	0	0	0	0	0	0	0	2	48
*M. avium*	18	8	1	1	0	1	0	1	0	0	0	0	0	0	30
*M. gordonae*	20	4	0	0	0	1	1	0	0	0	0	0	0	0	26
*M. kansasii*	9	4	0	2	1	0	0	0	0	0	0	0	1	0	17
*M. fortuitum*	13	1	0	0	0	0	1	0	0	0	0	0	0	0	15
*M. gilvum*	2	0	0	0	0	0	0	0	0	0	0	0	0	0	2
*M. xenopi*	2	0	0	0	0	0	0	0	0	0	0	0	0	0	2
*M. marinum/M. ulcerans*	0	0	0	0	1	0	0	0	0	0	0	0	0	0	1
*M. smegmati*	0	0	0	0	0	0	0	0	1	0	0	0	0	0	1
*M. terrae*	1	0	0	0	0	0	0	0	0	0	0	0	0	0	1
*M. phlei*	1	0	0	0	0	0	0	0	0	0	0	0	0	0	1
Other *Mycobacterium*	7	0	0	0	0	0	0	0	0	0	0	0	0	1	8
Total	154	30	9	8	7	4	2	1	1	1	1	1	1	3	223

## Discussion

Non-tuberculous mycobacteria (NTM) do not cause tuberculosis or leprosy, but can cause pulmonary disease resembling tuberculosis, lymphadenitis, skin disease, or disseminated disease. The pulmonary infection caused by NTM is difficult to clinically differentiate from that caused by MTB. However, most of the NTM strains are not susceptible to anti-tuberculosis drugs. The treatment plan is related to the species, infection site and the severity of infection [9]. Therefore, rapid and accurate identification of *Mycobacterium* to species level is very important for managing these infections. PCR-fluorescence probe method (Tuberculosis and Non-Tuberculous Mycobacteria Real-time PCR Detection Kit, CapitalBio Technology Inc., Beijing, China) can be used to detect both MTB or NTM in the specimen directly in 3 hours, and there is no significant difference in the detection performance of this method compared to traditional methods (culture and microscopy) [10]. In addition, the DNA microarray chip method (Mycobacterial Species Identification Array Kit, CapitalBio Technology Inc., Beijing, China) can detect and identify 17 species or groups of clinically common *Mycobacteria*, including *M. tuberculosis* complex, *M. intracellular*, *M. avium*, *M. gordonae, M. kansasii*, *M. fortuitum*, *M. scrofulaceum*, *M. gilvum*, *M. terrae*, *M. chelonae /M. abscessus*, *M. phlei*, *M. nonchromogenicum*, *M. marinum* /*M. ulcerans*, *M. aureus, M. senegalense / M. malmoense*, *M. xenopi* and *M. smegmati*. Using this method, results are available within 6 h, which is helpful for early diagnosis and treatment of tuberculosis and NTM disease.

An analysis of the current study in relation to previous findings showed that the absolute numbers of both NTM and MTB positive samples increased yearly from 2013 to 2018. Notably, the proportion of NTM positive samples among the total positive samples for MTB and/or NTM as per the PCR-fluorescence probe method (Tuberculosis and Non-Tuberculous Mycobacteria Real-time PCR Detection Kit, CapitalBio Technology Inc., Beijing, China), increased from 15.6% in 2013 to 46.1% in 2018. This is in agreement with the results of the national tuberculosis epidemiological sampling surveys carried out in China in 1990 (4.9%), 2000 (11.1%) and 2010 (22.9%) [11, 12]. This finding is consistent with those from multiple studies in diverse countries, demonstrating an increasing proportion of NTM infections in recent years [13, 14]. Many factors may contribute to the observed rise in NTM detection, including improved clinical awareness of NTM infections, use of better and more sensitive NTM detection techniques, increased number of immunocompromised patients, aging of the population, the threshold to test a sample, and alteration of sample types for NTM detection [15, 16]. As shown in Fig. 1, as the number of samples submitted for MTB or NTM identification increased, so did the positivity rate for NTM species whilst that of MTB decreased. Besides, as shown in Table 3, the number of samples tested for NTM from cerebrospinal fluid (CSF) and lymph node has increased several times over the last 3 years. In addition, the NTM positive rate in CSF or lymph node sample is just lower than in sputum samples but higher than in any other samples. The increase in the numbers of CSF and lymph node samples tested may explain the observed increase in the number of NTM positive samples. Among the most common NTM pathogens, the incidence of MAC grew faster than that of *M. chelonae /M. abscessus*, from 2013 to 2018 (Table 1), suggesting a more prominent role for MAC in NTM infections. Furthermore, the fast increase in the proportion of *M. gordona* and M. *fortuitum* in NTM infections should be noted.

**Table 3:** The numbers of each different sample type tested for mycobacteria

Sample types	No. of tested samples (%)						
	2013	2014	2015	2016	2017	2018	Total
Sputum	402 (46.8)	626 (33.7)	954 (42.0)	1482 (45.5)	1940 (47.0)	2271 (46.2)	7675 (44.4)
Tracheobronchial aspiration	160 (18.6)	524 (28.2)	570 (25.1)	587 (18.0)	765 (18.5)	793 (16.1)	3399 (19.7)
Cerebrospinal fluid	57 (6.6)	109 (5.9)	155 (6.8)	358 (11.0)	452 (10.9)	697 (14.2)	1828 (10.6)
Bronchoalveolar lavage fluid	76 (8.8)	209 (11.3)	184 (8.1)	216 (6.6)	292 (7.1)	323 (6.6)	1300 (7.5)
Hydrothorax	57 (6.6)	90 (4.8)	116 (5.1)	175 (5.4)	194 (4.7)	207 (4.2)	839 (4.9)
Urine	43 (5.0)	82 (4.4)	93 (4.1)	127 (3.9)	141 (3.4)	194 (4.0)	680 (3.9)
Lymph node	3 (0.3)	36 (1.9)	32 (1.4)	128 (3.9)	104 (2.5)	149 (3.0)	452 (2.6)
Ascitic fluid	16 (1.9)	56 (3.0)	46 (2.0)	85 (2.6)	81 (2.0)	92 (1.9)	376 (2.2)
Pus	22 (2.6)	24 (1.3)	32 (1.4)	35 (1.1)	46 (1.1)	64 (1.3)	223 (1.3)
Others	23 (2.7)	101 (5.4)	89 (3.9)	66 (2.0)	115 (2.8)	121 (2.5)	515 (3.0)

The major clinical manifestation of NTM is pulmonary disease, with MAC the most common species involved in infection [17, 18]. In this retrospective study, *M. intracellulare* (65/193, 33.7%) was the most commonly detected species in respiratory tract samples, followed by *M. chelonae / M. abscessus* (37/193, 19.2%), *M. avium* (27/193, 14.0%)*, M. gordonae* (24/193, 12.4%). So in this study, MAC (92/193, 47.7%) was still the most common NTM pathogen in respiratory tract, which is consistent with previous findings in Europe, US, Canada, Australia, Japan, Korea, Southern China and so on [15–20]. Although MAC is the most common pathogen in NTM pulmonary disease, its relative frequency varies widely among different geographical regions. Many factors such as climate type and population density can affect the distribution of NTM species [2].

In the present study, NTM infection was much more common in women (55.2%) than men (44.8%). Furthermore, the age range of infected people was relatively wide, being most common in the 45–65 year age group (49.6%), probably due to some issues related to the function of the immune system [21]. This finding is in agreement with those of multiple studies in US [22], Japan [23, 24] and South Korea [20], which all indicated that older women were more susceptible to NTM infection. According to a previous study, abnormal expression of adipokines, sex hormones, and/or TGF-β may predispose slender, older women to NTM infection [25]. However, contrasting findings have been reported in Europe, where patients with NTM pulmonary disease were more likely to be male, possibly owing to smoking history and increased incidence of chronic obstructive pulmonary disease (COPD) [13, 24].

DNA microarray chip method (Mycobacterial Species Identification Array Kit, CapitalBio Technology Inc., Beijing, China) can accurately distinguish between *M. avium* and *M. intracellulae*, which have quite similar phenotypes. We found out that the prevalence of *M. intracellulae* (31.8%) was always higher than *M. avium* (13.5%) from 2013 to 2018. It is important to distinguish between *M. avium* and *M. intracellulae* because they show different pathogenic characteristics. *M. intracellulae* is more virulent compared to *M. avium,* indicating the need for a more intensive therapeutic strategy [26]. However, the DNA microarray chip method (Mycobacterial Species Identification Array Kit, CapitalBio Technology Inc., Beijing, China) could not distinguish between *M. chelonae* and *M. abscessus,* and between *M. marinum* and *M. ulcerans*. This is because *M. chelonae* and *M. abscessus* have the same 16S rRNA gene sequences, and so are *M. marinum* and *M. ulcerans*. However, *M. chelonae* tends to cause disseminated infections [9], and *M. ulcerans* produces a cytotoxin (mycolactone) with immune-modulating properties that causes necrosis [27]. Therefore, accurate identification of these strains to species level still has an important clinical significance. Microbiology laboratories could further identify these strains by 16S–23S Internal Transcribed Spacer Region sequencing and Sequencer-Based Capillary Gel Electrophoresis [28]. Moreover, Mycobacterial Interspersed Repetitive Unit-Variable Number Tandem Repeat (MIRU-VNTR) markers can be used for typing *M. intracellulae* clinical isolates for molecular epidemiological studies [29]. Other than pulmonary infectious diseases, NTM can also cause lymph node and skin and soft tissue infections. In this study, lymph node and pus were the second most common specimen types, only less than respiratory tract infections.

This study has several limitations. First, most NTM strains are widely distributed in the environment, being found in the soil, and water, including even treated water. Therefore, caution must be exercised in interpreting positive results from specimens as this doesn’t necessarily mean that the patient is infected by the bacteria. Positive results may be due to bacterial colonization, or transient infection which is quickly cleared by the immune system, or contamination in sample collection and transportation [18]. Second, the resolution power of our NTM detection method is limited. The Mycobacterial Species Identification Array Kit (CapitalBio Technology Inc., Beijing, China) cannot differentiate the following organisms into species level: *M. chelonae* and *M. abscessus, M. marinum* and *M. ulcerans, M. szulgai and M. malmoense* [30]. In addition, the kit cannot discriminate NTM strains into sub-species level. Over recent years, researchers have modified the sub-species taxonomies of many NTM species [31, 32]. The sub-species of *M. abscessus*, for instance, contains *M. massiliense* and *M. bolletii*, which are distinct in susceptibility to macrolides based on differences in the *erm* gene, thus leading to different therapeutic strategies if identified properly [32]. Third, among all the NTM-positive samples collected (565), only 40% (223) were further subjected to species identification testing. This was because some patients were lost to follow up, and some patients could not afford to pay for further NTM species identification. As a result, our data may not contain the overall NTM species information.

Finally, among the common NTM species, MAC, *M. abscessus* and *M. kansasii* are highly pathogenic, and can cause lung diseases and lymph node, skin and soft tissue infections. However, *M. gordonae* and *M. fortuitum* rarely cause any infections, and only cause diseases when the patient’s general condition is particularly poor. Thus during the clinical diagnosis and treatment, the clinical significance of the strain should be considered by combining with the patient’s symptoms, signs and imaging findings.

## Conclusions

The proportion of NTM strains among mycobacterial species detected in a tertiary hospital in Beijing, China, increased rapidly from year 2013 to 2018. Middle-aged and elderly patients are more likely to be infected with NTM, especially females. The most frequently detected NTM pathogens were *M. intracellulare* and *M. chelonae* / *M. abscessus*. Accurate and timely identification of NTM is crucial for diagnosis and treatment.

## Methods

### Data collection

This was a retrospective study. Clinical data of patients who tested positive/negative for *Mycobacterium tuberculosis* (MTB) or NTM using a screening test (Real-time fluorescent PCR detection) from January 2013 to December 2018 in Peking Union Medical College Hospital, Beijing, China, were retrospectively collected. The study was approved by the Human Research Ethics Committee of Peking Union Medical College Hospital (no. S-K890). Data on Mycobacteria species identification, which was carried out by DNA microarray chip, was also collected. To avoid bias in our retrospective analysis, only one specimen per body site of an individual was included in the current study.

### PCR-fluorescence probe method

#### Nucleic acid extraction

Tuberculosis and Non-Tuberculous Mycobacteria Real-time PCR Detection Kit (CapitalBio Technology Inc., Beijing, China) were used to extract nucleic acid from the specimens directly and thereafter Real-time fluorescent PCR for MTB or NTM screening was performed following the manufacturer’s instructions. Nucleic acid extraction procedures were adapted according to the methodology described elsewhere [33]. Briefly, the mixture of 4% NaOH solution and the specimen (1:1) was vortexed and then incubated at room temperature for 30 min. A 1 mL aliquot of this suspension was centrifuged at 12000 rpm for 5 min. The supernatant was discarded and 50 μL of nucleic acid extract fluid was added to the pellet, vortexed thoroughly, and then put into Extractor™ 36 Nucleic Acid Extractor (CapitalBio Technology Inc., Beijing, China) and centrifuged at maximum rotational speed for 5 min. The tube was subsequently put in a 95 °C metal bath for 5 min, centrifuged at 5000 rpm for 1 min, and then the supernatant could be preserved at (− 20 ± 5) °C for 1 month.

#### Real-time fluorescent PCR detection

PCR amplification system was performed according to the manufacturer’s instructions of Tuberculosis and Non-Tuberculous Mycobacteria Real-time PCR Detection Kit (CapitalBio Technology Inc., Beijing, China) [10] and was roughly as follows; 18 μL PCR amplification reagent and 2 μL template DNA were mixed together; amplification conditions: 37 °C for 5 min, 94 °C for 3 min, and followed by the amplification of 40 cycles of 94 °C for 15 s, 60 °C for 30 s, then 50 °C for 10 s. FAM and HEX channels were selected for fluorescence detection at the same time. 60 °C for 30 s was the fluorescence signal acquisition point. A result with a Ct value of < 40 was considered positive. Negative and positive quality control products were included in parallel with the samples. Interpretation of real-time fluorescent PCR assay results is shown in Table 4.

**Table 4:** Interpretation of real-time fluorescent PCR assay results

Detection results	Interpretation
FAM (+) HEX(+)	MTB nucleic acid detection positive
FAM (+) HEX(−)	MTB nucleic acid detection positive
FAM (−) HEX(+)	NTM nucleic acid detection positive
FAM (−) HEX(−)	*Mycobacterium* spp. nucleic acid detection negative

*MTB Mycobacterium tuberculosis*, *NTM* Non-tuberculous mycobacteria

#### DNA microarray chip method

DNA microarray chip from Mycobacterial Species Identification Array Kit (CapitalBio Technology Inc., Beijing, China) was performed for NTM species identification as previously reported [30, 34]. Briefly, 9 μL hybridization buffer and 6 μL PCR products were mixed; reaction conditions: 95 °C for 5 min, ice bath for 3 min, then blowing and mixing. After this 13.5 μL of hybridization reaction mixture was added into the sampling hole, and then the hybridization box was sealed to maintain a 50 °C constant temperature in the water bath pot for 2 h. Then washing and drying of the chip followed, and then scanning with LuxScan 10 K-B Microarray Scanner (CapitalBio Technology Inc., Beijing, China). The corresponding software was used to read the signals and display the results.

### Statistical analyses

Excel was used to establish the database, and SPSS 22.0 was used for statistical analysis. Trend analysis of annual constituent ratio was carried out by trend Chi-square tests and a *P* value < 0.01 was considered statistically significant.

## Data Availability

The datasets analyzed during the current study are not publicly available due to the privacy of patients but are available from the corresponding author on reasonable request.

## References

[1] Honda JR, Knight V, Chan ED (2015). Pathogenesis and risk factors for nontuberculous mycobacterial lung disease. Clin Chest Med.

[2] Zhang ZX, Cherng BPZ, Sng LH, Tan YE (2019). Clinical and microbiological characteristics of non-tuberculous mycobacteria diseases in Singapore with a focus on pulmonary disease, 2012-2016. BMC Infect Dis.

[3] Hu C, Huang L, Cai M, Wang W, Shi X, Chen W (2019). Characterization of non-tuberculous mycobacterial pulmonary disease in Nanjing district of China. BMC Infect Dis.

[4] Porvaznik I, Solovic I, Mokry J (2017). Non-Tuberculous mycobacteria: classification, diagnostics, and therapy. Adv Exp Med Biol.

[5] Griffith DE, Aksamit T, Brown-Elliott BA, Catanzaro A, Daley C, Gordin F (2007). An official ATS/IDSA statement: diagnosis, treatment, and prevention of nontuberculous mycobacterial diseases. Am J Respir Crit Care Med.

[6] Tan Y, Su B, Shu W, Cai X, Kuang S, Kuang H (2018). Epidemiology of pulmonary disease due to nontuberculous mycobacteria in southern China, 2013-2016. BMC Pulm Med.

[7] Liu H, Lian L, Jiang Y, Huang M, Tan Y, Zhao X (2016). Identification of species of Nontuberculous mycobacteria clinical isolates from 8 provinces of China. Biomed Res Int.

[8] Hoefsloot W, van Ingen J, Andrejak C, Angeby K, Bauriaud R, Bemer P (2013). The geographic diversity of nontuberculous mycobacteria isolated from pulmonary samples: an NTM-NET collaborative study. Eur Respir J.

[9] Falkinham JO (2015). Environmental sources of nontuberculous mycobacteria. Clin Chest Med.

[10] Guo L, Xu Y, Sun H, Song H, Wang Y, Zhao Y (2015). [clinical application of real-time FQ-PCR assay in rapid detection of *Mycobacterium spp* infection] (in Chinese). Chin J Nosocomio.

[11] Technical Guidance Group of the Fifth National TB Epidemiological Survey (2012). [The fifth national tuberculosis epidemiological survey in China in 2010] (in Chinese). Chin J Antituberc.

[12] National Technical Steering Group of the Epidemiological Sampling Survey for Tuberculosis. [Report on nationwide random survey for the epidemiology of tuberculosis in 2000] (In Chinese). Chin J Antituberc. 2002;25(1):3–7. 11953089

[13] Prevots DR, Loddenkemper R, Sotgiu G, Migliori GB (2017). Nontuberculous mycobacterial pulmonary disease: an increasing burden with substantial costs. Eur Respir J.

[14] Donohue MJ, Wymer L (2016). Increasing prevalence rate of Nontuberculous mycobacteria infections in five states, 2008-2013. Ann Am Thorac Soc.

[15] Horne D, Skerrett S (2019). Recent advances in nontuberculous mycobacterial lung infections. F1000Res.

[16] Namkoong H, Kurashima A, Morimoto K, Hoshino Y, Hasegawa N, Ato M (2016). Epidemiology of pulmonary Nontuberculous mycobacterial disease, Japan. Emerg Infect Dis.

[17] Prevots DR, Marras TK (2015). Epidemiology of human pulmonary infection with nontuberculous mycobacteria: a review. Clin Chest Med.

[18] Stout JE, Koh WJ, Yew WW (2016). Update on pulmonary disease due to non-tuberculous mycobacteria. Int J Infect Dis.

[19] Brode SK, Marchand-Austin A, Jamieson FB, Marras TK (2017). Pulmonary versus nonpulmonary Nontuberculous mycobacteria, Ontario, Canada. Emerg Infect Dis.

[20] Lee H, Myung W, Koh WJ, Moon SM, Jhun BW (2019). Epidemiology of Nontuberculous mycobacterial infection, South Korea, 2007-2016. Emerg Infect Dis.

[21] Marras TK, Mehta M, Chedore P, May K, Al Houqani M, Jamieson F (2010). Nontuberculous mycobacterial lung infections in Ontario, Canada: clinical and microbiological characteristics. Lung..

[22] Adjemian J, Olivier KN, Seitz AE, Holland SM, Prevots DR (2012). Prevalence of nontuberculous mycobacterial lung disease in U.S. Medicare beneficiaries. Am J Respir Crit Care Med.

[23] Morimoto K, Iwai K, Uchimura K, Okumura M, Yoshiyama T, Yoshimori K (2014). A steady increase in nontuberculous mycobacteriosis mortality and estimated prevalence in Japan. Ann Am Thorac Soc.

[24] van Ingen J, Wagner D, Gallagher J, Morimoto K, Lange C, Haworth CS (2017). Poor adherence to management guidelines in nontuberculous mycobacterial pulmonary diseases. Eur Respir J.

[25] Chan ED, Iseman MD (2010). Slender, older women appear to be more susceptible to nontuberculous mycobacterial lung disease. Gend Med.

[26] Jang MA, Koh WJ, Huh HJ, Kim SY, Jeon K, Ki CS (2014). Distribution of nontuberculous mycobacteria by multigene sequence-based typing and clinical significance of isolated strains. J Clin Microbiol.

[27] Gehringer M, Altmann KH (2017). The chemistry and biology of mycolactones. Beilstein J Org Chem.

[28] Subedi S, Kong F, Jelfs P, Gray TJ, Xiao M, Sintchenko V (2016). 16S-23S internal transcribed spacer region PCR and sequencer-based capillary gel electrophoresis has potential as an alternative to high performance liquid chromatography for identification of slowly growing Nontuberculous mycobacteria. PLoS One.

[29] Dauchy FA, Degrange S, Charron A, Dupon M, Xin Y, Bebear C (2010). Variable-number tandem-repeat markers for typing *Mycobacterium intracellulare* strains isolated in humans. BMC Microbiol.

[30] Liu J, Yue J, Yan Z, Han M, Han Z, Jin L (2012). Performance assessment of the CapitalBio mycobacterium identification array system for identification of mycobacteria. J Clin Microbiol.

[31] Jagielski T, Borowka P, Bakula Z, Lach J, Marciniak B, Brzostek A (2019). Genomic insights into the *Mycobacterium kansasii* complex: an update. Front Microbiol.

[32] Tortoli E, Kohl TA, Brown-Elliott BA, Trovato A, Leao SC, Garcia MJ (2016). Emended description of *Mycobacterium abscessus*, *Mycobacterium abscessus* subsp. *abscessus* and *Mycobacterium abscessus* subsp. *bolletii* and designation of *Mycobacterium abscessus* subsp. *massiliense* comb. nov. Int J Syst Evol Microbiol.

[33] Guo Y, Zhou Y, Wang C, Zhu L, Wang S, Li Q (2009). Rapid, accurate determination of multidrug resistance in *M. tuberculosis* isolates and sputum using a biochip system. Int J Tuberc Lung Dis.

[34] Shi XC, Liu XQ, Xie XL, Xu YC, Zhao ZX (2012). Gene chip array for differentiation of mycobacterial species and detection of drug resistance. Chin Med J.

